# Interferon Response in Hepatitis C Virus-Infected Hepatocytes: Issues to Consider in the Era of Direct-Acting Antivirals

**DOI:** 10.3390/ijms21072583

**Published:** 2020-04-08

**Authors:** Pil Soo Sung, Eui-Cheol Shin

**Affiliations:** 1 Division of Gastroenterology and Hepatology, Department of Internal Medicine, College of Medicine, Seoul St. Mary’s Hospital, The Catholic University of Korea, Seoul 06591, Korea; 2 The Catholic Liver Research Center, College of Medicine, The Catholic University of Korea, Seoul 06591, Korea; 3 Graduate School of Medical Science and Engineering, Korea Advanced Institute of Science and Technology (KAIST), Daejeon 34141, Korea

**Keywords:** hepatitis C virus, interferon, innate immunity, direct-acting antivirals

## Abstract

When interferons (IFNs) bind to their receptors, they upregulate numerous IFN-stimulated genes (ISGs) with antiviral and immune regulatory activities. Hepatitis C virus (HCV) is a single-stranded, positive-sense RNA virus that affects over 71 million people in the global population. Hepatocytes infected with HCV produce types I and III IFNs. These endogenous IFNs upregulate a set of ISGs that negatively impact the outcome of pegylated IFN-α and ribavirin treatments, which were previously used to treat HCV. In addition, the *IFNL4* genotype was the primary polymorphism responsible for a suboptimal treatment response to pegylated IFN-α and ribavirin. However, recently developed direct-acting antivirals have demonstrated a high rate of sustained virological response without pegylated IFN-α. Herein, we review recent studies on types I and III IFN responses in HCV-infected hepatocytes. In particular, we focused on open issues related to IFN responses in the direct-acting antiviral era.

## 1. Introduction

Interferons (IFNs) have critical antiviral activities and immune regulatory functions in infections and autoimmunity. These IFN functions are mediated by inducing the expression of various IFN-stimulated genes (ISGs) [1]. IFNs are divided into three major types: type I, which includes IFN-α and -β species; type II, which includes IFN-γ species, and type III, which includes IFN-λ species [2]. Type III IFNs were not identified until nearly five decades after the discovery of type I IFNs. Types I and III IFNs are similar in their induction mechanisms and in the intracellular signaling pathways that lead to ISG expression. However, they use different receptors and are encoded by distinct genes [2]. The type III IFN family is composed of four members: IFN-λ1 (IL-29), IFN-λ2 (IL-28A), IFN-λ3 (IL-28B), and the recently identified IFN-λ4. Among these, IFN-λ4 is unique, because it is only produced in individuals with a ΔG allele of the variant rs368234815-ΔG/TT [3,4]. Type III IFN responses to infections or autoimmunity are predominantly observed in epithelial barriers, including the gastrointestinal and respiratory tracts [5,6,7,8]. Early IFN-λ antiviral activity limits infection and prevents triggering other systemic immune responses and inflammation [5,6]. However, the induction of a persistent type III IFN response can hamper cellular responses to type I IFNs, particularly IFN-α [2,9,10,11].

Globally, around 71 million individuals are infected with hepatitis C virus (HCV). HCV is a single-stranded, positive-sense RNA virus [12] with a 9.6 kb genome. HCV encodes structural proteins, including the core, E1, and E2 proteins; it also encodes non-structural proteins, including p7, NS2, NS3, NS4A, NS4B, NS5A, and NS5B [13]. Acute HCV infections tend to evolve to chronic persistent infections. Historically, HCV infections were generally treated with pegylated IFN-α (peg-IFN-α) and ribavirin. However, with the recent introduction of direct-acting antivirals (DAAs), which target various HCV non-structural proteins, patients can be treated without serious adverse effects, and a high rate of sustained virological response (SVR) can be achieved [13]. However, in some cases, HCV treatment is ineffective. One potential explanation for this result could be the presence of genotype- or subtype-specific resistance-associated substitutions that are either pre-existing or drug-induced [14].

HCV-infected hepatocytes produce types I and III IFNs, which leads to the prolonged upregulation of ISGs in HCV-infected livers [10,11,15,16,17,18,19,20,21]. Several research groups, including ours, have shown that HCV-infected liver cells produced predominantly type III IFNs [9,10,11,22]. Furthermore, we demonstrated that IFN-λ4 was rapidly produced in HCV-infected primary human hepatocytes that carried the rs368234815-ΔG allele, which encodes the fully active IFN-λ4 protein [9]. Previous studies have mainly focused either on the association between *IFNL4* single nucleotide polymorphisms (SNPs) and spontaneous HCV clearance or on the pegylated-IFN-α treatment response [3,23,24,25]. The *IFNL4* genotype has remained notable in the DAA era. It was recently shown that the *IFNL4* genotype affected the response to DAA-based regimens among patients with HCV [26,27,28].

This review covers the recent progress on types I and III IFN responses in hepatocytes and their roles in HCV infections. We focus on open issues associated with types I and III IFN responses that should be considered in the DAA era.

## 2. Canonical and Non-Canonical Types I and III IFN Signaling Pathways in Hepatocytes

### 2.1. Canonical Types I and III IFN Signaling

Types I and III IFNs initiate intracellular signaling through autocrine and paracrine pathways [2]. Types I and III IFNs bind to heterodimeric receptors, including type I IFN receptor 1/2 (IFNAR1/2) and IFN-λ receptor 1 (IFNLR1)/interleukin 10 receptor 2 (IL10R2), respectively [29]. When types I and III IFNs bind to their receptors, the intracellular receptor subunits undergo conformational changes to interact with Janus kinases (JAKs), which lead to the activation of the JAK/signal transducer and activator of transcription (STAT) pathway. The JAKs include JAK1, JAK2, JAK3, and tyrosine kinase 2 (TYK2) [30]. The binding of both type I and type III IFNs to their respective receptor complexes leads to the phosphorylation of JAK1 and TYK2. On the other hand, the binding of type II IFN (IFN-γ) to the receptor complex triggers the phosphorylation of JAK1 and JAK2 complex. These activated JAKs phosphorylate tyrosine residues on IFN receptors, which then recruit STAT proteins [29]. In activating JAK-STAT signaling, IFN-γ mainly stimulates the formation of STAT1 homodimers, which directly bind to DNA [31]. Alternatively, types I and III IFNs mainly drive the formation of STAT1-STAT2 heterodimers, which bind to interferon regulatory factor 9 (IRF9); together, these proteins form a complex known as interferon-stimulated gene factor 3 (ISGF3) [31]. ISGF3 is translocated to the nucleus, where it acts as a transcription factor to induce the expression of ISGs [29]. In the nucleus, STAT1 homodimers bind to the γ-activated sequence (GAS) on DNA, and ISGF3 binds to interferon-stimulated regulatory elements (ISREs) on DNA [31]. ISGs have upstream regulatory regions that carry different types of STAT-binding elements, including solitary GAS elements, solitary ISREs, or combined GAS/ISRE elements [31]. Moreover, diverse binding sites and transcription factors that are active in the regulatory regions upstream of ISGs can subtly shift to allow the expression of different ISG subsets [2]. Consequently, upon IFN stimulation, ISG expression can vary in composition, kinetics, and scale, depending on which IFNs induce the signaling cascade [2].

The degree of ISG expression differs significantly between types I and III IFNs. Type I IFN is typically more potent than type III IFN [7,11,31,32,33]. IFN-β is associated with the highest degree of ISG expression, followed by (in decreasing order): IFN-α, IFN-λ3, IFN-λ1, and IFN-λ2 [11,32]. Studies on ISG expression kinetics have shown that type I IFNs stimulated earlier ISG expression than type III IFNs in cells that expressed both IFNAR1 and IFNLR1 [7,11,31,32,33].

A recent study by Forero and colleagues demonstrated the mechanisms underlying IFN-stimulated expression kinetics and immune-modulating effects [34]. They highlighted the differences between type I IFN responses and type III IFN responses. Type I IFNs uniquely activated the transcription factor, interferon regulatory factor 1 (IRF1) [34]. When STAT1 homodimers were formed, IFN-α induced IRF1 activation. IRF-1 mediated chemokine production, which recruited cytotoxic lymphocytes and natural killer cells [34,35]. Furthermore, Forero and colleagues also emphasized that the failure of type III IFNs to induce IRF1-mediated chemokine production was due to low IFNLR1 expression levels. When IFNLR1 expression was forced, type III IFNs could induce IRF1-mediated chemokine production [33,34]. The importance of IRF1 to the antiviral reaction in liver cells was demonstrated in a different study [36]. That study revealed that an instant defense mechanism against viruses was initiated by a series of ISGs, and the basal transcription levels of those ISGs relied on the constitutive expression of IRF1 [36].

### 2.2. Non-Canonical Types I and III IFN Signaling

Although tyrosine phosphorylation is critical for STAT activation; unphosphorylated STAT proteins continue to perform integral functions that activate ISGs [37]. In recent studies, unphosphorylated STATs (U-STATs; i.e., those with unphosphorylated tyrosine residues) were observed in the nucleus, where they bound to the upstream regulatory regions and initiated the expression of a set of ISGs [10,11,31,38,39,40]. One study reported that stimulating cells with a low IFN-β concentration maintained ISG expression for two or three days after tyrosine-phosphorylated STAT1 reverted to U-STAT [38]. Another report demonstrated the role of unphosphorylated ISGF3 (U-ISGF3) in prolonged ISG induction with type I IFN treatment [39]. After initially stimulating cells with type I or III IFN, abundant quantities of STAT1, STAT2, and IRF9 were produced. Even without tyrosine phosphorylation, these proteins combined to form U-ISGF3 [11,31,39]. The unique binding of U-ISGF3 resulted in the expression of approximately 30 ISGs. The upregulation of these ISGs was durable; it persisted in cells for several days after the initial IFN stimulation [11,31,39]. The majority of ISGs induced by U-ISGF3 had antiviral activity, and they could protect against DNA damage [31,39]. Another recent study demonstrated that IFN-independent baseline ISG expression was mediated by the U-ISGF3 complex in hepatocytes, liver organoids, and liver tissue cultures [41]. In that study, a phosphorylation-deficient STAT1 mutant protein was also active and non-inferior to the wild-type protein in its ability to upregulate ISGs that conferred antiviral activity in liver-derived cells [41]. Interestingly, mice with a homozygous Y701F mutation in *Stat1* showed a dramatic reduction in Stat1 protein expression. That finding suggested that initial IFN-dependent signaling played an important role in Stat1 expression. The rapid induction of ISGs by types I and II IFNs was absent in phosphorylation-defective *Stat1-Y701F* cells [42,43].

Previous studies with *STAT1*-null cells showed that STAT1-independent, STAT2-dependent gene expression was delayed after stimulation with type I IFNs [44]. Intriguingly, phosphorylated STAT2 and IRF9 in *STAT1*-deficient cells could also guide an extended transcriptional response, which resembled the response produced by ISGF3 to induce antiviral effects [44]. In addition to the contribution of U-STAT2 to U-ISGF3 formation, U-STAT2 could also bind constitutively to a considerable number of IFN-activated promoters, even in the absence of exogenous IFN stimulation [45]. Those findings corroborated earlier studies, which demonstrated that STAT2 could mediate innate immune responses in the absence of STAT1 [37].

### 2.3. Regulation of Types I and III IFN Signaling

Excessive IFN stimulation is prevented through the close regulation of STAT activation by ubiquitin carboxy-terminal hydrolase 18 (USP18) and inhibitory regulators of STAT activities, including the family of protein inhibitors of activated STAT (PIAS) and suppressors of cytokine signaling (SOCS) [30,37]. USP18 is a 368-amino acid protein that typically functions as an ISG15 isopeptidase. USP18 is promptly induced by viral infection and IFN signaling [46]. USP18 interacts with IFNAR2 to prevent JAK1 from interacting with IFNAR2, and thus, USP18 suppresses signal transmission from IFN-α binding [47,48]. According to recent studies, USP18 protein stability is maintained by free intracellular ISG15 protein in human cells [11,49,50]. Another study showed that an ISG15 deficiency resulted in increased viral resistance in humans, but not in mouse models, because only human ISG15 could maintain USP18 stability by preventing its ubiquitination [50]. It must be noted that this process was independent of the ISGylation activity of ISG15 and the delSGylation activity of USP18 [49].

The JAK/STAT pathway is also negatively modulated by SOCS family proteins [51]. More specifically, signaling mediated by type I IFNs is primarily suppressed by the interplay between SOCS1 and TYK2 and by the binding of SOCS3 to JAK2 [51,52]. The PIAS family also includes negative regulators of IFN signaling. PIAS proteins function as small ubiquitin-like modifier (SUMO) E3 ligases, which interfere with STAT signaling functions [37,53]. Phosphorylation of the proximal tyrosine residue on STAT1 is prevented by PIAS-induced STAT1 SUMOylation, which results in the generation of partially phosphorylated STAT dimers. These partially phosphorylated STAT dimers negatively affect JAK/STAT pathway activation by competing with fully phosphorylated STAT dimers [54,55].

## 3. Types I and III IFN Responses in HCV Infections

### 3.1. Host Factors Involved in HCV Sensing and IFN Production

**Figure 1:**
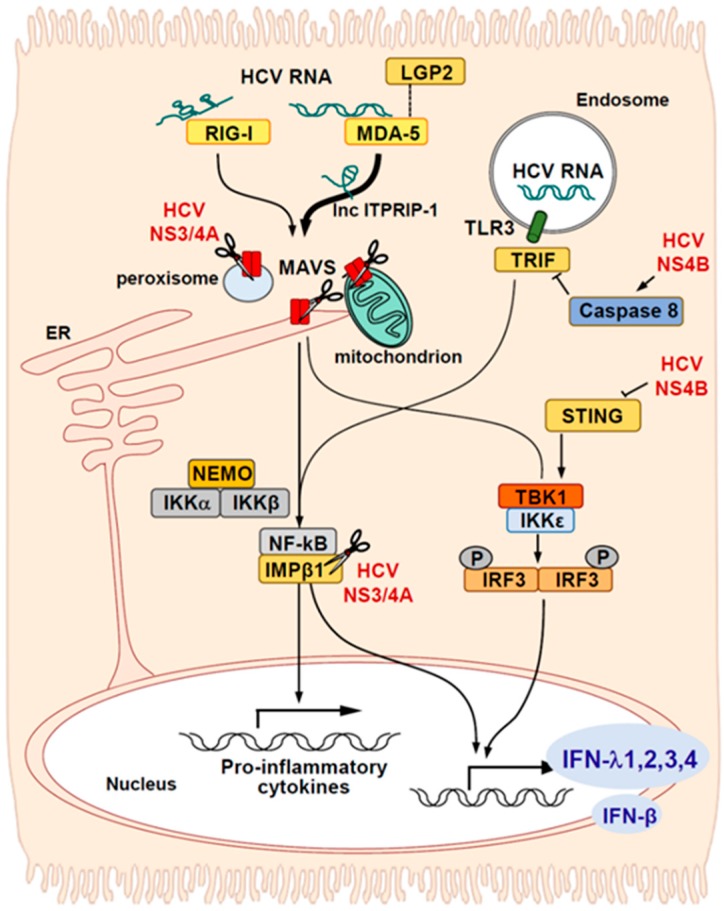
Induction of types I and III interferons (IFNs) in hepatitis C virus (HCV)-infected hepatocytes. After HCV enters hepatocytes, double-stranded RNA intermediates are recognized preferentially by MDA5 in the cytoplasm and by TLR3 in the endosome. LGP2 contributes to the efficacy of MDA5 in recognizing HCV RNA. HCV-induced lncRNA ITPRIP-1 stimulates the oligomerization and activation of MDA5. When MDA-5 binds to MAVS, they activate downstream NF-κB and IRF3 pathways, which lead to the induction of IFN-β, IFN-λs, and other pro-inflammatory cytokines. HCV NS3/4A protease (pictured as scissors) cleaves MAVS and partially disrupts the associated signaling. HCV NS3/4A protease also cleaves IMPβ1, which transports NF-κB from the cytoplasm to the nucleus. HCV NS4B activates caspase 8, which degrades TRIF and hampers the signal transduction from TLR3. NS4B also targets STING and blocks IRF3 phosphorylation. Despite these evasive HCV strategies, a considerable amount of IFNs are produced. Pointed arrows indicate activation; Blocked arrows indicate inhibition. IKKε: IkB kinase-ε.

IFN production and ISG upregulation occur in hepatocytes after HCV infections, although HCV interferes with this process (Figure 1) [10]. The production of endogenous IFNs is initiated by pattern recognition receptors (PRRs) in hepatocytes. There are three classes of PRRs: retinoic acid-inducible gene-I (RIG-I)-like receptors (RLRs), toll-like receptors (TLRs), and nucleotide-binding oligomerization domain-like receptors (NLRs) [56]. A previous study found that RIG-I detected the HCV 3′ untranslated region (UTR). However, a more recent study found that melanoma differentiation-associated protein 5 (MDA5), not RIG-I, played a central role in detecting HCV RNA [57,58]. Another study demonstrated a sequential activation of the innate signaling by RIG-I and MDA5 after HCV infection [59]. The binding of RIG-I or MDA-5 to mitochondrial antiviral-signaling protein (MAVS) resulted in the downstream activation of nuclear factor-kappa B (NF-κB) and IRF3 [60]. Recent evidence has suggested that IFN signaling triggered by HCV critically relies on a probable ATP-dependent RNA helicase, known as the laboratory of genetics and physiology 2 (LGP2) protein. LGP2 probably mediates MDA5 identification of HCV RNA [61]. Cellular HCV sensing is also facilitated by protein kinase R (PKR). PKR binding to HCV dsRNA prompted PKR to activate MAVS, which triggered ISG expression [62]. IFN expression and ISG upregulation were also induced when TLR3 recognized HCV RNA in endosomes [63,64]. A recent study in mice that lacked canonical NF-κB signaling showed that the hepatocyte-intrinsic NF-κB was a critical amplifier of type I IFN signaling; it induced ISG upregulation, the recruitment of activated immune cells, and viral clearance [65].

Many IFN-induced, long non-coding RNAs (lncRNAs) remain to be investigated [66]. One study demonstrated that the HCV-induced lncRNA, ITPRIP-1, stimulated MDA5 oligomerization and activation, which enhanced antiviral responses to HCV infections (Figure 1) [67]. Another study revealed that the antiviral innate immune response was controlled by lncRNA-IFI6 [68]. This lncRNA modulated the expression of IFN-α-inducible protein 6 (IFI6), by activating its promoter and modifying its histones. These activities were mediated through the lncRNA spatial domain [68]. Notably, HCV infections were regulated by IFI6, regardless of whether the JAK-STAT pathway was activated [68].

### 3.2. Viral Evasion from Endogenous IFN Responses in HCV-Infected Cells

In general, viruses evade IFN regulation with diverse mechanisms. Like other viruses, HCV partially disrupts the induction of IFNs in infected cells (Figure 1) [69]. The HCV NS3/NS4A protease cleaves MAVS at Cys-508, independent of whether it is localized to mitochondria-associated endoplasmic reticulum membranes [70] or peroxisomes [71]. The cleavage of MAVS by the HCV serine protease was verified by analyzing HCV-infected liver tissues [72]. Interestingly, a conserved MAVS mutation found in non-human primates prevented viral protease antagonism and resulted in unchecked IFN production in ‘resistant’ MAVS-expressing cells [73]. HCV NS4B can also inhibit RLR-mediated IFN production by targeting a protein called the stimulator of interferon genes (STING), an adaptor protein that facilitates IRF3 phosphorylation by TANK-binding kinase 1 (TBK1) [74,75,76]. However, the mechanisms which dictate how NS4B blocks the STING-mediated signaling pathway are different among the published reports. Ding et al. demonstrated that NS4B disrupts the binding of STING and TBK1 [76], whereas Nitta et al. showed that NS4B blocks the interaction between MAVS and STING [75]. The third report showed that genotype 2a NS4B suppressed the accumulation of STING protein in a dose-dependent manner, although the exact mechanism was not described [74]. Earlier studies reported that the HCV serine protease cleaved the protein called toll/IL-1 receptor domain-containing adaptor, inducing IFN-β (TRIF), which is the downstream mediator of TLR3-induced signal transduction [77]. However, recent observations could not support that finding. A recent study found that NS4B protein downregulated TRIF at the protein level through a unique mechanism [64]. They showed that NS4B activated caspase 8 to enhance TRIF degradation, which resulted in the suppression of TLR3-mediated IFN signaling [64]. NS3/NS4A protease also degrades importin β1 (IMPβ1), which transports proteins from the cytoplasm to the nucleus [78]; this activity leads to impaired NF-kB p65 trafficking between the cytoplasm and nucleus.

### 3.3. Type III IFNs in HCV-Infected Hepatocytes

Although HCV inhibits IFN production in infected hepatocytes, type III IFN production persists in HCV-infected cells. As a result, IFN-λs are expressed at high levels in livers with chronic HCV infections [10,79,80]. Several studies have demonstrated, in cell culture models, that IFN-λs were expressed at higher levels than IFN-βs in HCV-infected cells (Figure 1) [9,11,22,81]. Later, it was found that only cells that are infected with HCV express IFN-λs; uninfected cells near the infected cell do not express IFN-λs [82]. Mouse hepatocytes do not express IFNLR1 protein and are not responsive to IFN-λs. In contrast, IFNLR1 is expressed at high levels in human hepatocytes, and IFN-λs play an important role in resisting HCV and other viral infections [35]. IFN-λs are generated and function as long as HCV persists in the liver; thus, the infected liver exhibits high expression levels of many ISGs [10,11,81,82]. Numerous ISGs were found to play various roles during the HCV life cycle [83]. ISGs restrict the viral spread and induce various types of immune responses [83].

The efficacy of IFN-λ in HCV clearance in vitro and the limited pattern of IFNLR1 expression in humans led to an interest in the use of IFN-λ for treating hepatotropic viral infections. Consequently, pegylated IFN-λ was developed for treating HBV or HCV infections. Clinical trials that evaluated the safety and efficacy of pegylated IFN-λ for treating HCV infections showed promising results [84]. Moreover, adverse effects associated with therapeutic IFN-λ might be less severe than those associated with type I IFNs, because fewer cell types express IFNLR compared to IFNAR, though both are expressed in hepatocytes. The clinical development of pegylated IFN-λ as a therapeutic option for chronic HCV infections was terminated upon the discovery of DAAs. However, clinical studies on pegylated IFN-λ highlighted the safety of pegylated IFN-λ, which raised the possibility that it may be useful for treating other viral infections in humans.

### 3.4. Impact of IFNL4 Genotype on the Outcome of HCV Infection

As mentioned earlier, humans and chimpanzees infected with HCV exhibit the continuous upregulation of ISGs in the liver [10,11,15,16,17,18,19,20,21]. Furthermore, ISG mRNAs were detected concurrently with HCV RNA in HCV-infected hepatocytes [85]. Despite the continuous upregulation of ISGs, livers that are chronically infected with HCV exhibit scant levels of phosphorylated STAT1 [11]. Instead, unphosphorylated STATs are evident in HCV-infected livers [39,41]. In HCV-infected cells, U-ISGF3 induces the expression of a particular set of ISGs, with antiviral activities that are constitutively sustained throughout the infection [11,41]. Prolonged ISG expression is not always beneficial; both spontaneous viral clearance [23] and SVR induced with IFN-α-based treatments [19,86,87] were negatively correlated with the upregulation of baseline ISGs [10,11]. A study from our group indicated that U-ISGF3 induced sustained ISG15 upregulation, as a consequence of sustained exposure to the IFN-λs produced endogenously during HCV infections [11]. Unresponsiveness to exogenous IFN-α therapy arose from the stabilization of USP18 protein by ISG15 [9,11,49]. Thus, the abundance of ISG15 in HCV-infected livers explained why patients with elevated baseline hepatic ISGs showed poor responses to IFN-α-based therapy [11,15,88].

The *IFNL4* gene was first identified in 2013 [89]. A germline dinucleotide frameshift variant in exon 1 of *IFNL4* gave rise to the expression of IFN-λ4 protein [89]. The full-length IFN-λ4 protein is produced by the *IFNL4*-ΔG allele, but not by the *IFNL4*-TT allele, due to a premature stop codon [29,89]. All four type III IFN-λs (IFN-λ 1-4) are produced by a majority of people of African descent. In contrast, only IFN-λs 1-3 are produced by most people of European and Asian descent [90].

Research on IFN-λ4 has been hampered by the lack of efficient systems for recombinant IFN-λ4 production. Previously, forced expression of the *IFNL4*-ΔG allele failed to produce significant amounts of IFN-λ4 protein in mammalian cells [89]. Although recombinant IFN-λ4 could be purified from a bacterial expression system [4], our group recently developed an efficient method for recombinant IFN-λ4 production [91]. We introduced new N-glycosylation sites in the IFN-λ4 protein, and found that the variant proteins were produced efficiently in mammalian cells. Moreover, the N-glycosylated IFN-λ4 displayed more robust ISG-inducing activity compared to a recombinant IFN-λ4 produced with a bacterial system [91]. Several studies have reported that ISG upregulation in primary human hepatocytes (PHHs) and HepG2 cells could be induced by forced *IFNL4* gene expression, or treatment with a recombinant IFN-λ4 protein [9,89,92,93] that displayed antiviral activity against HCV [9,93,94]. Recombinant IFN-λ4 and IFN-λ3 induce similar ISG expression patterns [4,9], because both activate the JAK-STAT pathway by binding to the IFN-λ receptor [9,95]. Our group demonstrated that HCV infections induced IFN-λ4 expression at both the protein and mRNA levels in PHHs [9].

Previous studies revealed that there are in vivo and in vitro phenotypic differences between two IFN-λ4 variants, IFN-λ4-P70 and IFN-λ4-S70 [23]. Compared to the IFN-λ4-P70 variant haplotype, the IFN-λ4-S70 variant displayed diminished in vitro antiviral effects [23]. Moreover, recent genome-wide association studies on human and viral data from Caucasian patients with HCV genotype 3a infections demonstrated that the differential production and functions of IFN-λ4-P70 and IFN-λ4-S70 impacted the viral load and the variability of viral amino acids. This variability was observed in human leukocyte antigen (HLA)-restricted epitopes and throughout the viral polyprotein [96]. All these findings support the hypothesis that IFN-λ4 plays a critical role in controlling HCV infections.

**Figure 2:**
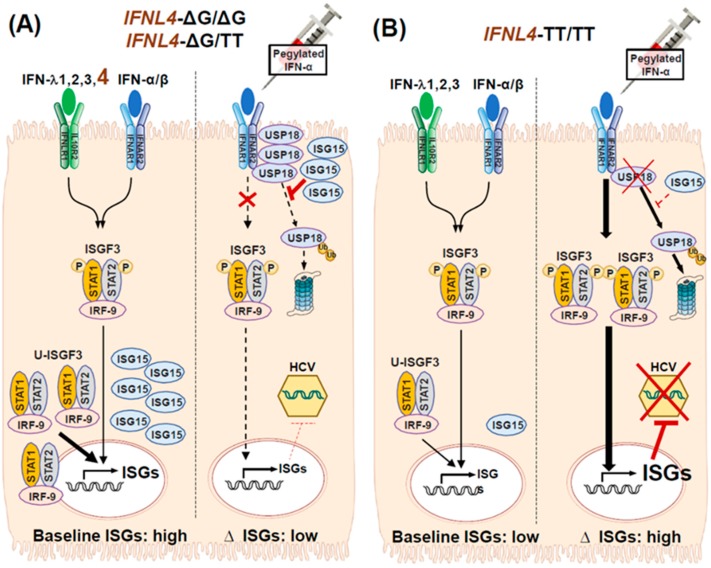
Roles of the *IFNL4* genotype in the responsiveness to exogenous IFN-α in HCV infections. (**A**) In patients with the *IFNL4*-ΔG/ΔG (rs368234815) or *IFNL4*-ΔG/TT genotype, IFN-λs (including IFN-λ4) and type I IFNs are produced. When type I and III IFNs bind to their receptors, they trigger the initial formation of ISGF3, which is composed of phosphorylated STAT1, phosphorylated STAT2, and IRF9. ISGF3 stimulates the expression of ISGs. After an HCV infection is established, ISG expression is maintained by U-ISGF3, which is composed of high levels of unphosphorylated STAT1, unphosphorylated STAT2, and IRF9. U-ISGF3 induces the abundant production of ISG15 in HCV-infected cells. Subsequently, ISG15 stabilizes the USP18 protein. In turn, USP18 blocks signaling through the IFN-α-bound IFNα/β receptor, which attenuates the response to exogenous IFN-α. As a result, pegylated IFN-α treatment has low efficacy. (**B**) In patients with the *IFNL4*-TT/TT (rs368234815) genotype, functional IFN-λ4 protein is not produced. In this case, U-ISGF3 is less abundant, which leads to weak ISG15 induction and low USP18 protein levels in infected hepatocytes. Consequently, the response to exogenous IFN-α is not attenuated. As a result, pegylated IFN-α has potent antiviral efficacy. Pointed arrows indicate activation; Blocked arrows indicate inhibition. Ub: ubiquitin.

Despite its antiviral activities, functional IFN-λ4 has a negative impact on IFN-α-based HCV treatments (Figure 2) [90]. A suboptimal treatment response to pegylated IFN-α in HCV infections was primarily associated with the *IFNL4*-ΔG allele [3,25,89]. Previous studies have demonstrated that the *IFNL4*-ΔG genotype was correlated with high intrahepatic ISG levels [9,10,97] and IFN-λ4 expression was correlated with high liver ISG15 levels in chronic HCV infections [18]. A functionally impaired form of the IFN-λ4 protein, the IFN-λ4-S70 variant, was associated with the relatively weak induction of intrahepatic ISGs; this finding indicated that IFN-λ4 was the primary driver of ISG inductions in HCV-infected livers [23]. In livers with the *IFNL4*-ΔG genotype, elevated baseline ISGs were associated with less additional ISG upregulation when treated with pegylated IFN-α (Figure 2). Thus, IFN-λ4 protein production in *IFNL4*-ΔG livers might play a critical role in upregulating ISG expression in chronic HCV infections. Our group previously demonstrated that both ISG15 and USP18 protein levels were elevated in HCV-infected livers [11]. We found that the prolonged stimulation of recombinant IFN-λ4 or transfection of the *IFNL4* gene induced robust, sustained ISG15 upregulation through the induction of U-ISGF3, which stabilized the USP18 protein and led to the unresponsiveness to exogenous IFN-α treatment (Figure 2) [9,11,22]. Thus, the *IFNL4* rs368234815-∆G allele hampered the response to pegylated IFN-α treatment.

A recent study examined whether *IFNL4* rs368234815 genotypes modulated ISG expression in peripheral blood mononuclear cells (PBMCs) during chronic HCV infections [98]. That study found that ISG expression was high in unstimulated PBMCs from patients homozygous for the unfavorable *IFNL4*-ΔG variant. Those findings suggested that *IFNL4* genotypes might have broader systemic effects during HCV infections [98]. Furthermore, *IFNL4* genotypes were associated with the metabolic alterations associated with HCV infections. One study showed different changes in low-density lipoprotein (LDL) levels, which depended on the *IFNL4* genotype of patients with chronic hepatitis C (CHC) [99]. LDL levels were elevated during DAA treatment in patients with CHC that achieved a successful SVR. Subsequently, LDL levels diminished, but only in patients that carried the *IFNL4*-ΔG gene, not in patients that carried the *IFNL4*-TT/CT gene [99]. Similarly, a different study showed that the *IFNL4* rs12979860 genotype was closely correlated with the levels of several types of lipids, including total cholesterol, LDL, and apolipoproteins [100].

## 4. Impact of DAA Treatment on Types I and III IFN Responses to HCV Infections

DAAs have significantly improved the treatment outcomes for chronic HCV infections. Consequently, IFN-α-based therapies are no longer used worldwide. In this section, we describe how DAA treatment has changed endogenous types I and III IFN responses, and how the *IFNL* genotype affects the outcome of IFN-free DAA treatment.

Recently, our group demonstrated in vitro that, in HCV-infected PHHs, DAA treatment abrogated the production of IFN-λs, including IFN-λ4, and restored responsiveness to exogenous IFN-α [9]. Other recent studies demonstrated that DAA treatment immediately downregulated ISG expression in the liver and blood, even in cases where prior IFN treatment was unsuccessful and in livers with high ISG expression [15,97,101,102]. Those findings were consistent with observations from a chimpanzee model of HCV infection, which showed that administration of a microRNA-122 antagonist rapidly reduced ISG levels in the liver [103]. Another recent study characterized the intrahepatic immune changes observed after curing HCV with DAA therapy [15]. That study compared ISG expression in non-tumor livers in patients with HCC, treated with or without DAAs. They demonstrated that, after DAA-based therapy, viral clearance resulted in reduced ISG expression, but the immune-cell profiles were similar between the two groups [15].

As previously discussed, HCV infections cannot be eradicated by endogenous type III IFN activities and, in many chronic infection cases, HCV replicates in the presence of elevated ISG levels. The rs368234815-ΔG allele, which encodes the fully active IFN-λ4, was correlated with the upregulation of ISGs in infected livers. Recently, an association was also observed between the *IFNL4* genotype and virologic relapse after DAA therapy [16,26,28]. The recent BOSON trial tested patients infected with HCV genotype 3 that were treated with sofosbuvir and ribavirin. They revealed that the *IFNL4*-CC genotype was closely correlated with SVR and with lower baseline ISG levels in peripheral blood and liver [16]. Another recent analysis of the ION-3 trial showed that the *IFNL4* rs12979860 polymorphism was associated with virologic relapse in patients infected with HCV genotype 1, after eight weeks of sofosbuvir and ledipasvir treatment [27]. Compared to patients with the unfavorable *IFNL4* rs12979860-CT or TT genotype, fewer patients with the *IFNL4* rs12979860-CC genotype experienced a viral relapse (5.8% vs. 1.8%) [27,104]. In the 8-week arm of the POLARIS-2 trial, the probability of relapse after sofosbuvir, velpatasvir, and voxilaprevir treatment was five-fold higher in patients with the unfavorable *IFNL4* rs12979860 genotype (CT or TT), compared to patients with *IFNL4* rs12979860-CC [26,105]. The favorable *IFNL4* rs12979860-CC and rs368234815-TT/TT genotypes were detected more frequently in people of Asian descent than in those of European descent; thus, Asian individuals might have markedly low virologic relapse rates after DAA treatment [26].

In contrast, a recent report showed that patients responsive to DAA treatment exhibited higher levels of intrahepatic ISGs at baseline than patients that experienced a viral breakthrough [97]. Consistent with that study, another study reported that the frequency of Y93H, the NS5A resistance-associated substitution, in HCV genotype 1 was closely correlated with the presence of favorable *IFNL4* genotypes and a high viral load at baseline. This finding might explain why *IFNL4* genotypes were not correlated or were negatively correlated with treatment responses to DAA regimens that included NS5A inhibitors [106]. Another study showed that intrahepatic ISG expression at treatment conclusion was higher with a SVR than with a viral relapse [101]. Based on that study, we could infer that maintaining intrahepatic type I IFN signaling at the end of treatment, and not ISG downregulation, might facilitate HCV eradication and the prevention of viral relapse after DAA treatment [101]. Consistent with that study, another recent report showed that DAA plus ribavirin treatment upregulated the expression of one ISG, called tripartite motif-containing 22 (TRIM22) [107]. They observed that TRIM22 expression was higher in patients that achieved SVR compared to untreated patients [107]. Future research should investigate the precise role of *IFNL4* genotypes in determining treatment responses to DAA regimens.

DAA therapy also rapidly restored the activated type I IFN response in PBMCs, assessed in terms of the ISG expression level and STAT1 phosphorylation [102,108,109]. A recent study from our group demonstrated that pre-treatment ISG activation in PBMCs might be due to type I IFN activation of JAK-STAT signaling [108]. Another recent study demonstrated that, at two and four weeks after starting DAA treatment, most ISGs were downregulated in PBMCs, and at the end of treatment, they were relatively elevated, but not above the baseline ISG level [102]. Interestingly, the serum levels of IFN-α-related chemokines increased concurrently with the reduction in ISG levels, but Th1 and Th2-related cytokines did not show that behavior [102]. In another study, among patients with HIV-1 that had acute HCV infections, ISGs were downregulated in PBMCs during DAA therapy. After therapy, the ISG levels increased in patients that relapsed, but not in patients that achieved a SVR [109]. In patients with HCV infections, baseline ISG levels in PBMCs were affected by the *IFNL4* genotype [98]. Furthermore, the unfavorable *IFNL4* genotype was encountered more frequently in HCV infections, when patients had cryoglobulinemic vasculitis and metabolic extrahepatic symptoms. That finding suggested that DAA therapy might alleviate HCV-induced extrahepatic symptoms by downregulating extrahepatic ISGs [110,111].

## 5. Conclusions

Worldwide, DAAs remain either unavailable or cost-prohibitive to a considerable proportion of the population of patients with HCV infections in developing countries. Moreover, although DAAs are highly effective, HCV cannot be cleared in some difficult-to-treat patients. Understanding the HCV-induced IFN responses in the DAA era might facilitate the development of strategies for providing DAAs that are more accessible and more easily-administered. This understanding might also contribute to developing novel host-targeting agents that can be combined with DAAs.

Numerous aspects of virus-induced IFN induction and JAK/STAT pathway activation remain to be addressed. HCV and other RNA viruses tend to employ multiple strategies for evading IFN responses. We need a comprehensive understanding of these viral strategies to block viral persistence. To understand downstream IFN signaling, we need further clarification of the association between HCV-induced canonical and non-canonical JAK/STAT pathways. Moreover, the differential role of types I and III IFN responses in HCV-infected hepatocytes should be clarified. Clarifying the hepatocyte-specific immune responses to HCV infection, in association with the host *IFNL4* genotypes, may help researchers understand the initial defense mechanisms to other RNA viruses, such as SARS-CoV-2. Finally, IFN-λ4 seems to impact both the response to IFN-α-based treatment and the response to DAA treatment; these features of IFN-λ4 should be studied further and extended to other types of viral infections.
